# Influence of Sc Microalloying and Low-Frequency Electromagnetic Casting on the Microstructure and Properties of As-Rolled 7A36 Aluminum Alloy

**DOI:** 10.3390/ma18214899

**Published:** 2025-10-26

**Authors:** Honglei Liu, Lingfei Yang, Jiangpeng Liu, Wenzhu Shao, Xiangjie Wang

**Affiliations:** 1 School of Materials Science and Engineering, Harbin Institute of Technology, Harbin 150001, China; 2 China Northeast Light Alloy Co., Ltd., Harbin 150060, China; 3 Key Laboratory of Electromagnetic Processing of Materials, Ministry of Education, Northeastern University, Shenyang 110819, China; 4 Engineering Research Centre of Advanced Materials Preparing Technology, Ministry of Education, Northeastern University, Shenyang 110819, China

**Keywords:** micro-alloying Sc, LFEC, microstructure, properties, 7A36 aluminum alloy

## Abstract

This study examines the microstructure and properties of 7A36 aluminum alloys processed through low-frequency electromagnetic casting (LFEC) with microalloyed Sc. Following secondary hot deformation, the addition of Sc refined the average grain size from 3.8 μm to 0.9 μm and reduced the deformed texture content. In the T6-aged condition, the combined application of Sc and LFEC enhanced the hardness (234 HV to 238 HV), ultimate tensile strength (647 MPa to 693 MPa), and elongation (EL). Fractographic analysis revealed brittle fracture modes dominated by cleavage, with minor intergranular contributions. The corrosion resistance was poorest in the rolled state and superior in the two-stage-aged state. Under two-stage aging, the maximum corrosion depth for the Sc-modified, LFEC-processed alloy decreased from 113.6 to 49.1 μm. The synergistic integration of Sc alloying and LFEC significantly improved both the mechanical properties and corrosion resistance.

## 1. Introduction

Al-Zn-Mg-Cu alloys with high Zn content (>10 wt.%) are pivotal structural materials in aerospace due to their exceptional specific strength, thermal processability, and wear resistance [1,2,3]. Increasing the Zn content not only enhances the alloy strength but also reduces the melting points, thereby improving the energy efficiency during processing [4,5]. However, conventional casting struggles to produce high-integrity super-high-strength Al-Zn-Mg-Cu alloys, as coarse grains (>100 μm), non-equilibrium eutectics (e.g., T-Al_2_Zn_3_Mg_3_ phases), and elemental segregation inevitably form, degrading the mechanical and corrosion properties [6,7]. In industrial production, grain refiners and mechanical stirring methods are frequently employed. However, unfavorable pollution melts were produced. Therefore, casting ingots in appropriate magnetic or electric fields, or a combination of the two, produces grain refining without polluting the melt, making electromagnetic stirring and vibration popular study issues [8,9].

To date, some studies have investigated the improvement of the microstructure of Al-Zn-Mg-Cu alloys, which affects the mechanical properties and corrosion resistance of Al-Zn-Mg-Cu alloys [10], such as heat treatment [11,12], addition of microalloying elements [13,14], or adoption of new casting processes [15,16]. The low-frequency electromagnetic casting (LFEC) technique was developed and has been utilized for several years with the deployment of an induction coil positioned outside the standard direct chill (DC) casting mold. Electromagnetic processing via LFEC refines microstructures through forced convection and dendritic suppression without contamination [17,18,19]. The addition of microalloying elements is an effective method. Microalloying with Sc induces dual effects: primary Al_3_(Sc,Zr) particles refine as-cast grains to ~50 μm, whereas coherent secondary Al_3_(Sc,Zr) precipitates (L1_2_ structure) inhibit recrystallization during thermomechanical processing [20,21]. In addition, adding Sc to an Al alloy greatly increases the nucleation tendency; the formation of coarse primary Al_3_(Sc,Zr) particles can refine the grain structure, and the extremely fine coherent secondary Al_3_(Sc,Zr) particles with the L_12_ structure can effectively inhibit recrystallization in molten aluminum [22]. To date, extensive research has been conducted on the effect of Sc on precipitation or LFEC processing in Al-Zn-Mg-Cu alloys, and few researchers have systematically reported the effect of both on the microstructure and properties during the entire artificial aging process after severe deformation in such a high Zn Al-Zn-Mg-Cu alloy. Previous research by the research group has shown that the application of Sc and low-frequency electromagnetic fields refined and dispersed the second-phase particles of 7A36 alloy thin plates, enhancing the mechanical and corrosion properties of the alloy. However, different deformation amounts result in different microstructure changes in the alloy. Therefore, It is urgently necessary to discuss the microstructure and property changes of 7A36 alloy thick plates by applying Sc and low-frequency electromagnetic fields from the aspects of macroscopic and microscopic microstructure changes [23,24,25]. The critical gaps persist: (1) the combined effects of Sc addition and LFEC on post-deformation microstructure evolution (e.g., texture, precipitate distribution, and grain boundaries) remain underexplored for Al-Zn-Mg-Cu alloys subjected to extruding/rolling; (2) the impact of LFEC application and Sc-induced macroscopic and microscopic microstructure changes on corrosion resistance, particularly regarding intergranular corrosion susceptibility, is rarely addressed.

Herein, we systematically investigated the role of Sc and LFEC in optimizing the mechanical and corrosion properties of the 7A36 alloy after secondary deformation and aging. Specifically, we focused on the microstructural evolution during deformation and aging, mechanical performance linked to dynamic recrystallization and texture development, and corrosion behavior governed by Sc-modulated grain boundary continuity and Al_3_(Sc,Zr) phase distribution. This study bridges fundamental gaps in processing high-Zn Al alloys for aerospace applications, where optimized strength-corrosion trade-offs are critical.

## 2. Experimental Section

### 2.1. Alloy Preparation

In this research, three distinct alloys were fabricated: A1 (the base alloy processed via DC), A2 (the base alloy with the addition of 0.012 wt.% Sc, also processed using DC), and A3 (the base alloy containing 0.013 wt.% Sc, produced through LFEC). For simplicity in discussion, the rolled sheets will be designated as R1, R2, and R3, correspondingly. Initially, pure aluminum was melted in a resistance furnace utilizing a stainless-steel crucible. Upon melting the aluminum ingot, copper was introduced. When the ingot’s temperature reached approximately 740–750 °C, Zn was incorporated, a covering agent was applied, and heating continued. The furnace temperature then rose to about 760 °C, at which point pure Mg, Al-20Si, Al-10Mn, Al-5Zr, Al-2Sc, and A1-5Ti-B master alloys were blended into the molten aluminum. This smelting procedure was carried out within an argon atmosphere to avoid oxidation. Following this, the molten alloy was moved from the smelting furnace into the crystallizer mold at 720 °C, and billets with a diameter of 192 mm and a length of 500 mm were prepared by conventional DC and LFEC processing, respectively. The frequency of the alternating electromagnetic field was 22 Hz, and the flow rate of the cooling water was kept constant during the casting process. Additionally, the chemical compositions of the 7A36 aluminum alloy utilized in the as-cast state have been analyzed using Inductively Coupled Plasma Atomic Emission Spectroscopy (Beijing Huake Yitong Analytical Instrument Co., LTD., Beijing, China). The detailed findings regarding these compositions can be found in Table 1, each sample was conducted five times under different electromagnetic field conditions. Subsequently, the ingots were homogenized in the furnace at 460 °C for 24 h and hot extruded to 12 mm at 430 °C.

**Figure 1:**
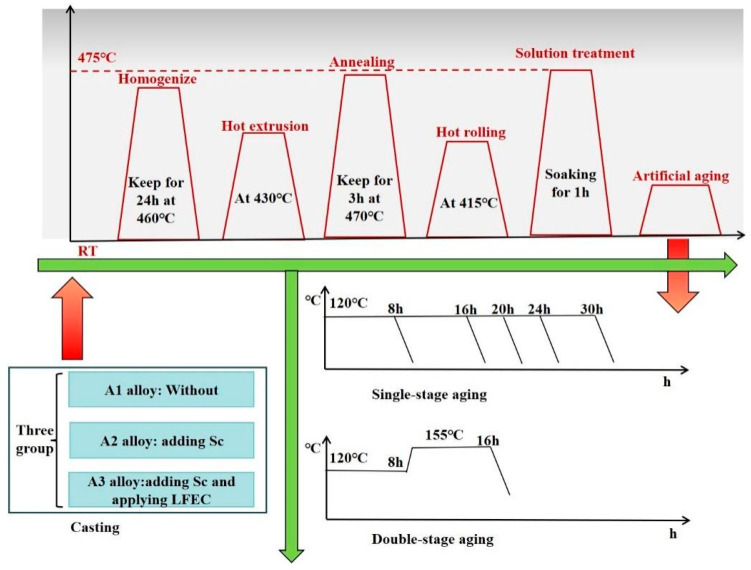
Scheme of the experimental process flow of the studied 7A36 aluminum alloy.

Then, the plates were annealed at 470 °C for 3 h and hot rolled into sheets with a thickness of 6 mm at an initial temperature of 415 °C. Finally, the alloy sheets were solution-treated at 475 °C for 1 h and quenched in water. Subsequently, they were aged in single- and double-stage aging treatments. The experimental process flow for the investigation of the 7A36 aluminum alloy is depicted in Figure 1. This diagram outlines the various stages and methodologies applied throughout the study.

### 2.2. Characterizations and Testing

**Figure 2:**
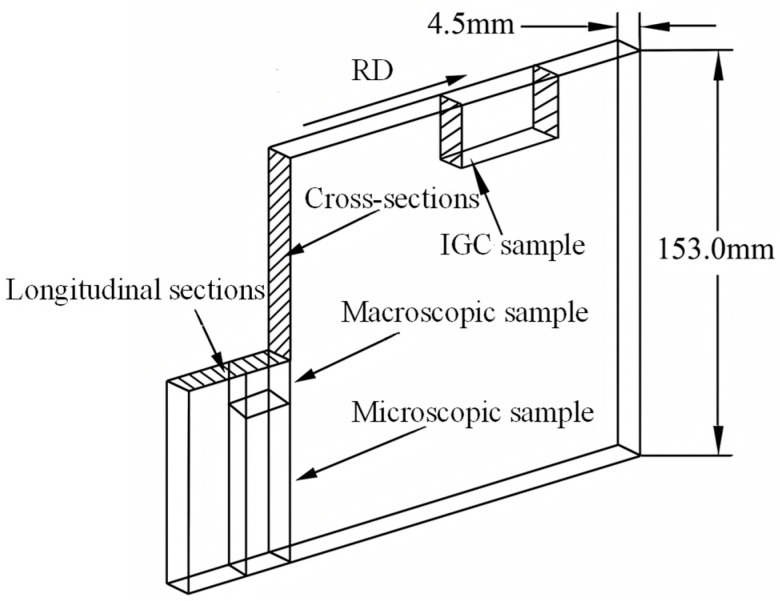
Sampling locations of as-rolled 7A36 aluminum alloy.

In this study, the 7A36 aluminum alloys, which were in their as-rolled condition, underwent several preparatory steps to facilitate microstructure observation. The samples were ground and polished meticulously to ensure a smooth surface for examination. Afterwards, they were subjected to anodizing treatment at a voltage of 20 V using a solution composed of 10 vol% HBF_4_. The rolling directions of the samples—namely the rolling direction (RD), the transverse direction (TD), and the normal direction (ND)—are illustrated in Figure 2. It is important to note that the samples selected for microstructure observation were specifically taken from regions along both the transverse direction and the normal direction to capture a comprehensive representation of the material’s structure. To gain deeper insights into the microstructural characteristics of the aluminum alloys, electron backscattering diffraction (EBSD) tests were conducted using Zeiss Crossbeam 550 microscopes which produced by Zeiss Company of Oberkochen, Germany. These tests were executed along the extrusion direction of the specimens, employing a fine step length of 0.23 μm to achieve high-resolution imaging of the microstructural features. The specimens were first ground by SiC paper to #3000 and then Argon ion polished to eliminate residual stress. To avoid spurious boundaries, a lower limit of 2° has been used in this investigation. The black and gray lines correspond to the high-angle grain boundaries (HAGBs) with misorientation more than 15° and the low-angle grain boundaries (LAGBs) with misorientation ranging from 2° to 15°, respectively, and the color of each grain is coded by its crystal orientation. In this experiment, the FM-700 microhardness tester produced by FUTURE-TECH Company of Kawasaki Japan was used to test the Vickers hardness of the samples. The minimum measurement unit was 0.1 μm, the load was 200 gf, and the holding time was 15 s. To further investigate the material’s characteristics, the electrical conductivity (EC) was determined with a Sigmascope EX 8 instrument at a controlled room temperature of 25 °C. The measurement’s accuracy is one significant digit to the decimal place, and the frequency of measurement is three times. Subsequently, the alloy sheets that had undergone aging were precisely machined into standard metal tensile specimens, adhering to the specifications outlined in GB/T 228.1-201 [26]. The gauge length and test speed of the specimens are 25 mm and 2 mm/min. To validate the results and enhance the reliability of the findings, each tensile test was conducted three times under each electromagnetic field condition. Following these repetitions, the average values for yield strength (YS), ultimate tensile strength (UTS), and elongation (EL) were calculated. This rigorous approach to testing allows for a robust analysis of the material’s performance under different conditions, providing valuable insights into its mechanical properties. In accordance with the specifications outlined in the standard GB/T 7998-2005 [27], testing for intergranular corrosion (IGC) was performed. This involved the application of solutions comprising 59 g/L NaCl and 10 mL/L H_2_O_2_ maintained at a temperature of 35 ± 1 °C. The samples were immersed in a water bath for a total of 6 h, with a minimum of three specimens being evaluated for each experimental condition. Following the immersion phase, the samples underwent cleaning and drying before being cut at a right angle to the RD. The specimens were subsequently ground and polished in alignment with the established methodology. The ratio of the solution to the sample surface area was designated as 25 mL/cm^2^. The measurement of corrosion depth was carried out using a Leitz MM-6 optical microscope.

## 3. Results and Discussion

### 3.1. Macro and Microstructure of As-Rolled 7A36 Aluminum Alloy

**Figure 3:**
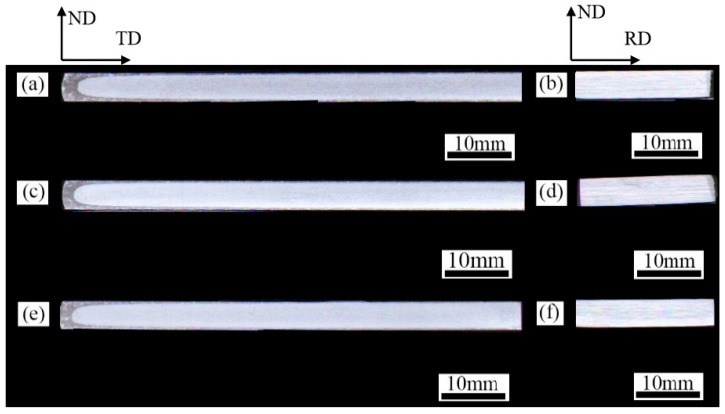
Macroscopic corrosion structure of as-rolled 7A36 aluminum alloy: (**a**,**b**) R1 alloy; (**c**,**d**) R2 alloy; (**e**,**f**) R3 alloy.

Figure 3 presents the transverse and longitudinal sections of the three groups of as-rolled 7A36 aluminum plates after macro-etching. Delamination was evident across all sections of the three aluminum alloy rolled plates, with the most pronounced occurrence observed near the edges of the transverse sections. Minor delamination was also observed on the upper and lower surfaces of the plates.

**Figure 4:**
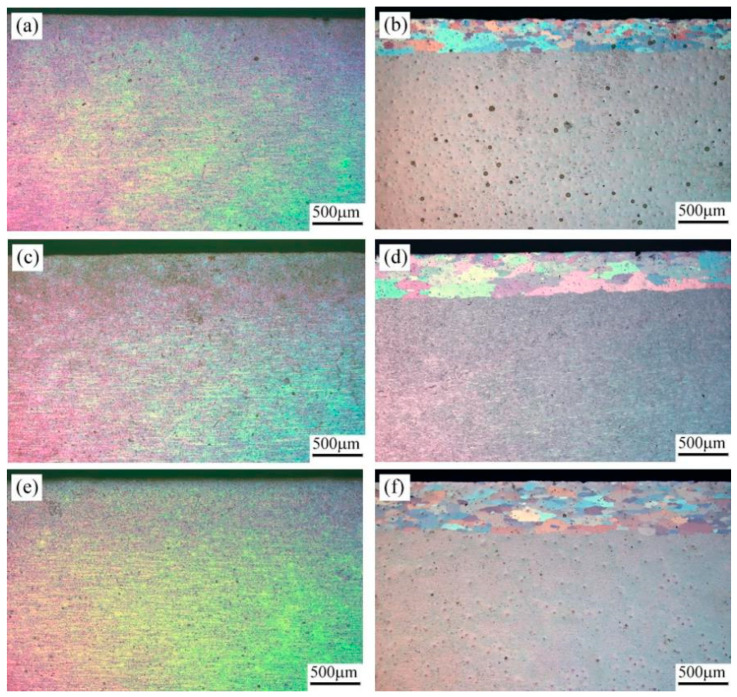
Anode coating structure of as-rolled 7A36 aluminum alloy in different states: (**a**,**b**) R1 alloy; (**c**,**d**) R2 alloy; (**e**,**f**) R3 alloy.

Figure 4a,c,e display the anodic oxide films for the as-rolled 7A36 aluminum alloy. The core regions of all the as-rolled sheets exhibited a fibrous microstructure. Figure 4b,d,f show the corresponding anodic oxide films after the aging treatment. Coarser grains appeared on the upper and lower surfaces of the rolled sheets, accompanied by numerous fine dark precipitates within the grains. The disappearance of the fibrous structure near the sheet edges is attributed to severe grain fragmentation and recrystallization resulting from the higher deformation levels experienced in these regions during hot-rolling. Following solution treatment, the microstructure evolves through two effects: (1) recovery and recrystallization-induced grain growth occurs at the edges owing to the elevated temperature [28], and (2) a supersaturated solid solution forms owing to rapid quenching. Subsequent aging promoted the precipitation of primary strengthening phases, predominantly η′ (MgZn_2_), leading to the observed high density of fine dark precipitates.

### 3.2. Effects of Sc and LFEC on the Microstructure and Texture Evolution of As-Rolled 7A36 Aluminum Alloys

**Figure 5:**
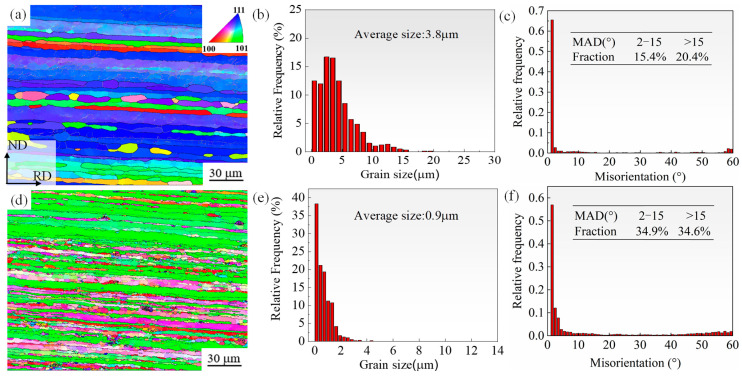
EBSD orientation micrographs, average grain size, and MAD histogram of as-rolled 7A36 aluminum alloy: (**a**–**c**) R1 alloy; (**d**–**f**) R2 alloy.

Figure 5 shows the EBSD orientation micrographs, grain size, and MAD histogram of the R1 and R2 alloys. The preferred orientation of the grains of the R1 alloy changed to a certain extent after hot-rolling deformation. The polycrystalline grain orientation was concentrated in the <111> direction, the grains were elongated owing to severe deformation in the hot-rolling process, and more broken grains and grain substructures appeared. After adding Sc, the grains became more uniform, and Al_3_(Sc_x_Zr_1−x_) particles formed during the pre-casting and subsequent homogenization processes, which hindered the movement of grain boundaries and inhibited the occurrence of recrystallization. Thus, the grains of the R2 alloy were significantly refined, and the LAGBs increased. This is due to the grain splitting caused by hot rolling deformation and a certain number of LAGBs are produced, in addition, the LAGBs of R2 alloy has increased, the second phase particles such as Al_3_(Sc_x_Zr_1−x_) precipitated during the hot rolling process of the 7A36 aluminum alloy after the elements play a role in inhibiting recrystallization and hinder the transformation of LAGBs to HAGBs, so that the sheet retains a relatively high Poly-sub crystalline structure. Compared with the R1 alloy, the grain size of the R2 alloy was significantly reduced from 3.8 μm to 0.9 μm, and the grain size distribution became relatively more uniform.

**Figure 6:**
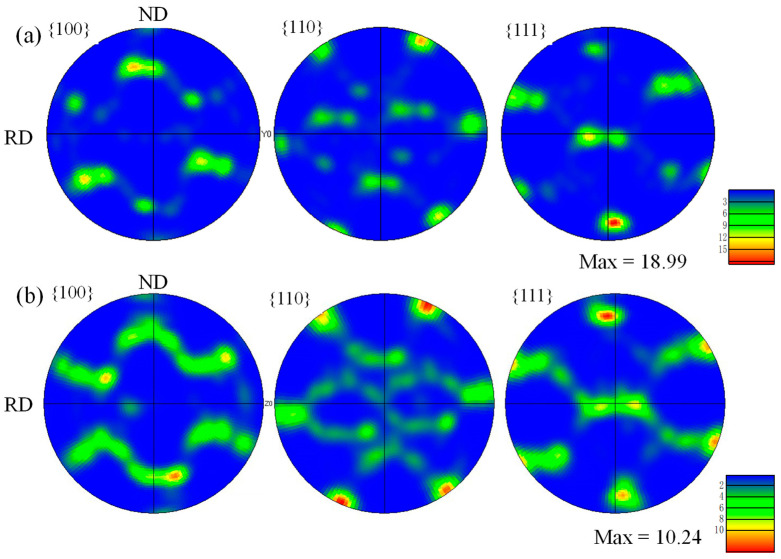
Pole figure of the as-rolled 7A36 aluminum alloy: (**a**) R1 alloy; (**b**) R2 alloy.

Figure 6 shows the {100}, {110}, and {111} pole figures of the as-rolled 7A36 aluminum alloy. After hot rolling, compared with the R1 alloy, the type and strength of the texture of the R2 alloy changed to a certain extent. Adding Sc resulted in an increase in the texture content of the 7A36 as-rolled aluminum alloy to a certain extent, whereas the strength decreased. The maximum density values of the R1 and R2 alloys were 18.99 and 10.24, respectively.

**Figure 7:**
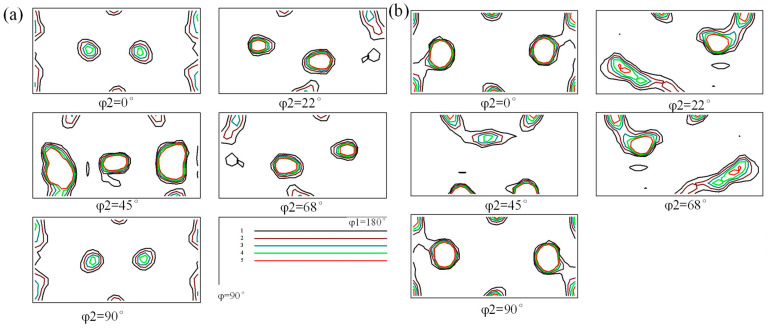
ODF mapping of as-rolled 7A36 aluminum alloy: (**a**) R1 alloy; (**b**) R2 alloy.

To further analyze the changing trend of the texture type and content of the as-rolled 7A36 aluminum alloy after hot rolling deformation. Figure 7 shows the orientation distribution function diagrams of the R1 and R2 alloys from 2° to 90°. The deformation textures in the sheet were mainly Brass{011}<211>, Copper{112}<111>, and S{123}<634> textures. The recrystallized texture was also dominated by R{124}<211>.

**Figure 8:**
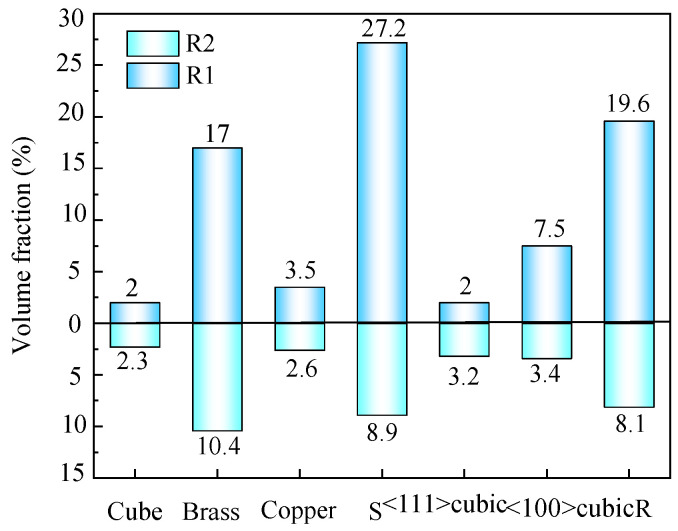
Texture content of as-rolled 7A36 aluminum alloy.

Figure 8 presents a statistical figure of the content of each texture type of the R1 and R2 alloys. After hot rolling, owing to the severe deformation of the sheet, the recrystallization texture shows a relatively low content, whereas the deformation texture content shows a relatively high content. Compared with the R1 alloy, the deformation texture content of the R2 alloy further decreased. It is possible that the precipitated phases, such as Al_3_(Sc_x_Zr_1−x_), generated after the addition of Sc, hinder the rotation of the crystal grains, adversely affecting the texture formation, and resulting in a significant decrease in the texture content [29]. After the addition of Sc, the recrystallization texture R{124}<211> in the rolled sheet decreased from 19.6% to 8.1%. This is because the addition of Sc inhibited recrystallization, which led to a decrease in the recrystallization texture.

### 3.3. Effects of Sc and LFEC on the Mechanical Properties of As-Rolled and As-Aged 7A36 Aluminum Alloys

**Figure 9:**
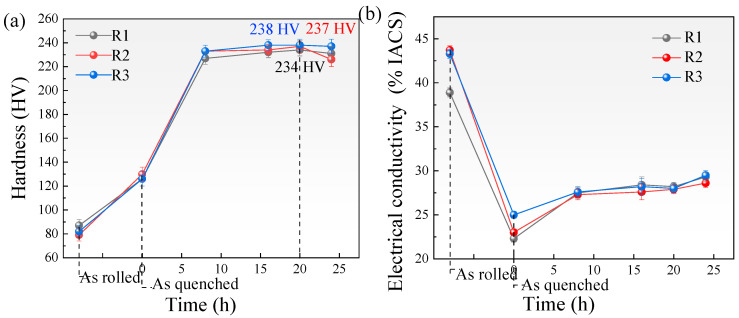
Hardness and EC change in as-rolled and as-aged 7A36 aluminum alloy. (**a**) Hardness; (**b**) EC.

Figure 9 shows the hardness and EC test results of the as-rolled 7A36 aluminum alloy in different aging states. The hardness of the as-rolled 7A36 aluminum alloy was relatively low, and after solution treatment, the hardness of the 7A36 aluminum alloys was significantly improved. This is because the second phase existing in the as-rolled undergoes a large amount of re-dissolution. A supersaturated solid solution was formed, and the hardness of the as-rolled sheet improved owing to the solid solution strengthening effect. With the extension of aging time, the hardness tends to first increase and then decrease. The hardness reached the highest value when aging at 120 °C for 20 h (T6). The hardnesses of the R1, R2, and R3 alloys were 234 HV, 237 HV, and 238 HV, respectively. After an aging time of more than 20 h, the hardness of the R1 and R2 alloys decreased significantly, and the hardness of the R3 alloy was relatively stable. This is because when the aging time is short, the high-density and fine GP zone is first precipitated from the aluminum matrix, so that the hardness of the sheet is significantly improved. After the aging time was extended, part of the GP zone began to dissolve, which caused the hardness of the sheet to decrease. The aging time continues to extend, and more GP zones begin to transform to the η′ phase, so the hardness begins to rise again, and the aging time is further extended. As the number of precipitated η′ phases reached a peak and began to coarsen, the hardness of the sheet no longer increased. There was even a downward trend.

After the T6 aging treatment, the hardness of the R2 alloy was higher than that of the R1 alloy. It is possible that after the addition of Sc, fine Al_3_(Sc_x_Zr_1−x_) precipitates with the L_I2_ cubic lattice structure gradually precipitated during the aging treatment. These precipitates are mainly distributed in the grain boundary and sub-grain boundary, and have a strong pinning effect, which can effectively hinder the movement of the grain boundary, thereby restricting the growth of the crystal grains and significantly improving the hardness of the sheet [30,31]. The EC of the 7A36 aluminum rolled alloy was the highest. After solution treatment and aging at 120 °C, the EC of the 7A36 aluminum rolled alloy exhibited an overall upward trend. When the aging time is reach 24 h, the EC was highest, the EC of R1, R2, and R3 alloys was 29.3%IACS, 29.5%IACS, and 28.6%IACS, respectively.

**Figure 10:**
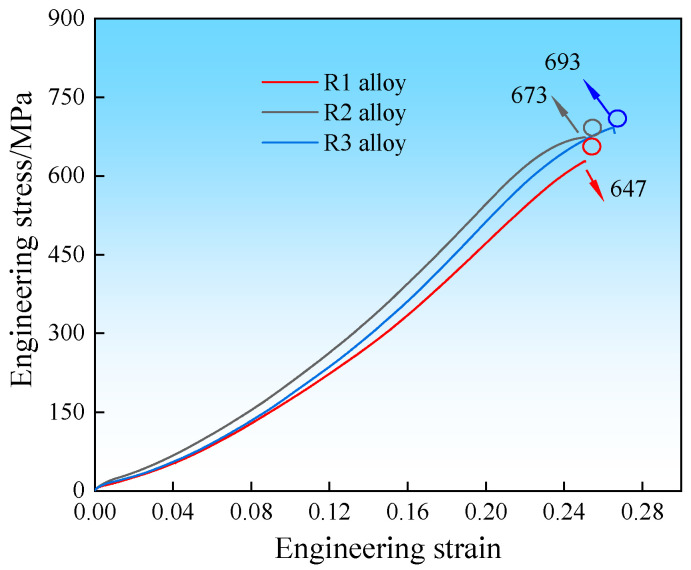
Tensile properties of as-aged 7A36 aluminum alloy.

To investigate the effects of Sc and LFEC on the strength of the 7A36 aluminum alloy, tensile testing was performed. This is because only slight recrystallization occurred in the hot-rolling process, and the dispersoids, such as MgZn_2_ (η phase), hindered the movement of the grain boundaries and the formation of the subgrain structure [32]. Thus, work hardening plays a major role in hot rolling processing. As shown in Figure 10, the typical engineering stress–strain curves of the 7A36 aluminum alloys in the T6 state showed that, compared with the R1 and R2 alloys, the R3 alloy exhibited a higher UTS, which reached 693 ± 5 MPa, whereas the UTS of the R1 and R2 alloys was 647 ± 5 MPa and 673 ± 8 MPa, respectively.

Generally, Sc is added to the Al matrix to act as a recrystallization inhibitor. During the solution process, the material dissolves into the aluminum (Al) matrix, resulting in the formation of a saturated solid solution. However, this particular type of solid solution is inherently unstable and tends to dissolve back out of the matrix during the subsequent homogenization stage. Within the aluminum matrix, certain dispersed compounds, specifically Al_3_(Sc_x_Zr_1−x_), precipitate out, contributing to the microstructural properties of the alloy. The hot-rolling process leads to significant deformation of the sheet, which in turn creates LAGBs. Upon the addition of Sc, the ratio of these LAGBs experiences a notable increase. This increase can be attributed to the presence of Al_3_(Sc_x_Zr_1−x_) particles situated at the grain boundaries, where they impede the movement of grains during the deformation process. Furthermore, during the initial casting procedure, the application of an electromagnetic field enhances the uniformity of both the composition and temperature distribution within the molten metal of the R3 alloy. The stirring effects induced by this electromagnetic field can effectively disrupt dendritic formations, elevate the nucleation temperature, and subsequently lead to a refinement of the grain size in the alloy. As a result of the subsequent hot deformation and heat treatment, a more uniform microstructure may be created, which aids in the hardening process [33,34]. On the one hand, the electromagnetic stirring effect generated by the site is conducive to the formation of the primary phase Al_3_(Sc_x_Zr_1−x_), which has a strong pinning effect on the grain boundary in the subsequent hot deformation process. However, it can effectively improve the alloy ingots. The inhomogeneity of the composition and temperature produced during the casting process further optimizes the microstructure, which is conducive to subsequent plastic deformation. The Al_3_(Sc_x_Zr_1−x_) phase can also inhibit recrystallization and grain growth, thereby playing a role in fine-grain strengthening and further improving the strength of the R3 alloy. In addition, the refined grain size enhanced the elongation of the R3 alloy to a certain extent.

**Figure 11:**
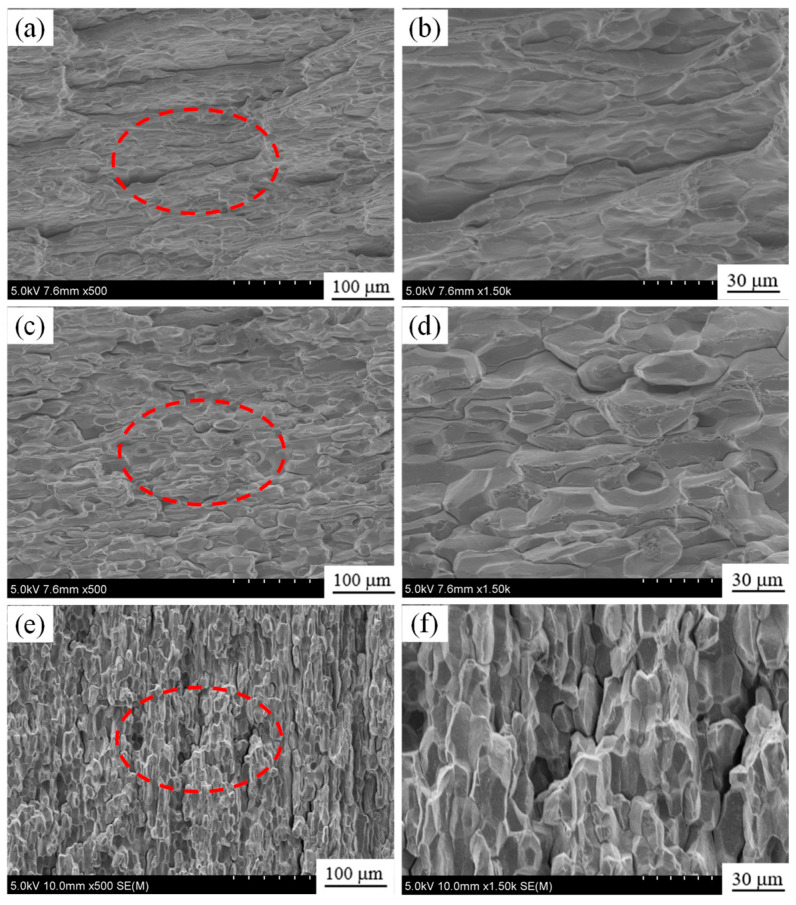
Tensile fractured surfaces of 7A36 aluminum alloys at T6 state: (**a**,**b**) R1 alloy; (**c**,**d**) R2 alloy; (**e**,**f**) R3 alloy; (**a**,**c**,**e**) Low-magnification SEM image of R1, R2 and R3 alloy, (**b**,**d**,**f**) Highmagnification SEM image of the red-framed zone in (**a**,**c**,**e**).

Figure 11 shows the tensile fracture of the three groups of as-rolled 7A36 aluminum alloys aged in the T6 state. The tensile fracture morphologies of the R1 and R3 alloys presented a rock sugar-like fracture, which is judged to be obvious characteristic of an intergranular fracture. The R2 alloy appears to be a cleavage step formed by small planes at different heights, and it is judged to be a cleavage fracture.

### 3.4. Effects of Sc and LFEC on the Corrosion Resistance As-Rolled 7A36 Aluminum Alloy

**Figure 12:**
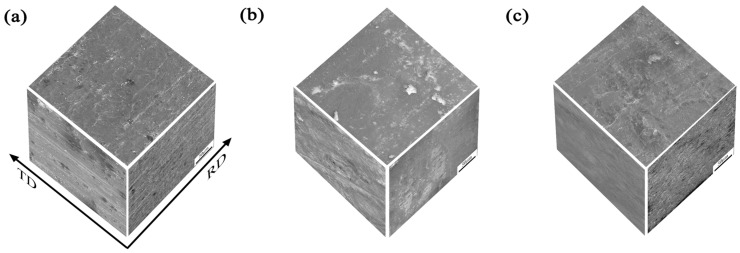
Three-dimensional scanning of as-rolled 7A36 aluminum alloys after corrosion in two-stage aging. (**a**) R1 alloy; (**b**) R2 alloy; (**c**) R3 alloy.

Figure 12 illustrates the surface morphologies of the three groups of as-rolled 7A36 aluminum alloys observed via SEM after double-stage aging treatment. Distinct variations in the intergranular corrosion morphology were evident across the different sheet orientations. The RD-TD plane exhibited minor features of exfoliation corrosion, characterized by irregular corrosion pits. In contrast, the RD-ND plane displayed pronounced corrosion grooves aligned with the RD, whereas the TD-ND plane exhibited a similar band-shaped corrosion morphology.

**Figure 13:**
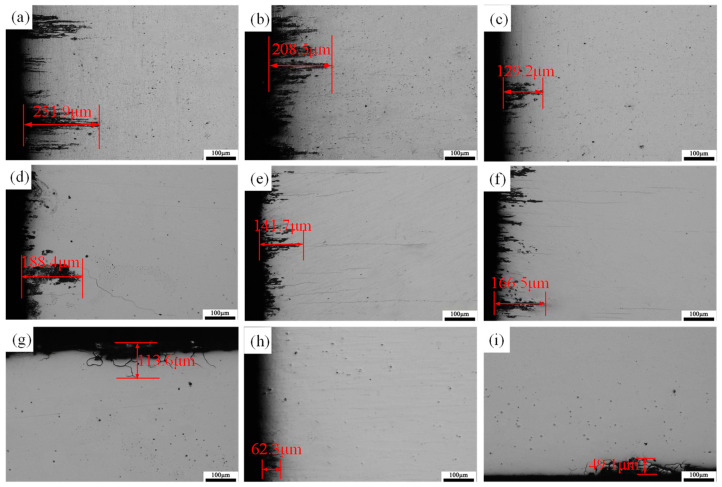
IGC diagram of 7A36 aluminum alloy: (**a**–**c**) as-rolled alloy; (**d**–**f**) single-stage aging treatment; (**g**–**i**) double-stage aging treatment.

To quantitatively assess the intergranular corrosion (IGC) resistance of as-rolled 7A36 aluminum alloys under different heat treatment conditions, the maximum IGC depth was examined using optical microscopy for specimens under each condition. Figure 13a–c present the IGC morphologies of the as-rolled 7A36 aluminum alloy. The R1 alloy exhibited the deepest maximum corrosion depth of 251.9 µm. The addition of Sc reduced the maximum depth to 208.5 µm, whereas the group with both Sc addition and LFEC application during casting showed a further reduction to 129.2 µm. Figure 13d–f displays the IGC morphologies of the 7A36 aluminum alloy after the single-stage aging treatment. Compared with the as-rolled alloy, all groups exhibited reduced maximum corrosion depths, with the group containing Sc and processed with LFEC reaching a depth of 166.5 µm. Figure 13g–i illustrate the IGC morphologies of the 7A36 aluminum alloy following double-stage aging treatment. These results demonstrate a significant improvement in IGC resistance in all groups. The maximum depths were 113.6 µm for the R1 alloy, 62.3 µm for the R2 alloy, and 49.1 µm for the R3 alloy, respectively. Compared with the as-rolled and single-stage-aged conditions, double-stage aging treatment significantly enhanced the IGC resistance of the as-rolled 7A36 aluminum alloy.

The 7XXX series aluminum alloys primarily undergo IGC via an electrochemical mechanism. The difference in electrode potential between the precipitate-free zones (PFZs) along the grain boundaries (or the grain boundary precipitates themselves), the matrix, and the adjacent solute-depleted zones causes the PFZs and grain boundary precipitates to act as anodes and dissolve [35,36]. The results presented above demonstrate that the addition of Sc significantly enhanced the IGC resistance of the rolled sheets. This improvement was attributed to the Al_3_(Sc_x_Zr_1−x_) phase precipitated upon Sc addition, whose electrode potential was close to that of the aluminum matrix. This proximity reduces the potential difference, thereby improving the corrosion resistance of the 7A36 aluminum alloys [37,38]. The heat treatment condition also significantly influenced the corrosion resistance of the 7A36 aluminum alloys. Taking the R1 alloy as an example, the maximum corrosion depth in the as-rolled condition was 251.9 µm. After single-stage aging, this depth decreased to 188.4 µm, and after double-stage aging, it was further reduced to 113.6 µm. Peak aging treatment improves corrosion resistance because the precipitation of the η’ phase and GP zones reduces the Zn and Mg content within the aluminum matrix, consequently increasing its electrode potential. This reduces the driving force for the IGC. [39]. The coarsening and discontinuous distribution of precipitates at grain boundaries play a significant role in enhancing corrosion resistance. In the process of double-stage aging, the elevated temperature utilized during the second aging stage facilitates the coarsening and spheroidization of the plentiful η’ metastable phase and η equilibrium phase precipitates situated at the grain boundaries. This thermal treatment not only results in an increase in the size of these precipitates but also in the spacing between them. Such changes contribute to a reduction in the potential differences that exist among the various phases present in the material. Furthermore, in terms of grain boundary characteristics, the addition of Sc not only refines the grains but also promotes the precipitation phases at the grain boundaries (such as MgZn_2_ phase) to become finer, more dispersed, and discontinuously distributed, thereby breaking the structure of continuous reticular precipitation phases at the grain boundaries. This structure can significantly reduce the potential difference between grain boundaries and within grains and lower the driving force for intergranular corrosion. Meanwhile, Sc helps increase the proportion of LAGBs. Compared with the randomly distributed large-angle grain boundaries, the low-angle grain boundaries have a more regular structure, lower energy, and thus lower corrosion sensitivity. After applying a low-frequency electromagnetic field, the intergranular corrosion depth of the alloy is further reduced [25]. This is because the stirring effect of the magnetic field during the solidification process makes the distribution of Sc elements more uniform, inhibits the recrystallization of the alloy, optimizes the size and distribution of the precipitated phases at the grain boundaries, and increases the proportion of low-angle grain boundaries, thereby blocking the corrosion channels and reducing the corrosion tendency and rate of the material.

## 4. Conclusions

This research examined how Sc microalloying and the application of LFEC influence the microstructure, texture development, and characteristics of both as-rolled and as-aged 7A36 aluminum alloys. The conclusions derived from this study are as follows:

1. After the secondary hot deformation of the 7A36 aluminum alloy, the transverse and longitudinal sections of the rolled plate showed delamination, with fine recrystallized grains at the edges and a fibrous structure at the core. After the addition of Sc, the average grain size decreased from 3.8 to 0.9 mm, the content of small-angle grain boundaries increased, and the content of deformed texture decreased. Among them, the content of Brass texture decreased from 17.3% to 10.4%. The S and R textures decreased from 27.2% and 19.6% to 8.9% and 8.1%, respectively.
2. After hot rolling and solution aging treatment, the hardness of the rolled plates increased. In the T6 aging state, the hardness of the alloy increased from 234 HV to 238 HV when Sc was added and a low-frequency electromagnetic field was applied. After the double-stage aging treatment, the hardness of the alloy decreased. However, after the regression and re-aging processes, the hardness increased again. The variation patterns of the EC and hardness were opposite.
3. The addition of Sc and the application of low-frequency electromagnetic fields enhanced the strength and elongation of the 7A36 aluminum alloy. In the peak aging state, the tensile strength of the alloy increased from 647 MPa to 693 MPa. The fracture modes were all brittle fractures, mainly cleavage fractures, mixed with a small number of intergranular fractures.
4. The heat treatment state significantly affects the corrosion resistance of the rolled plates. The corrosion resistance of the plates was the worst in the rolled state and the best in the double-stage aging state. In the two-stage aging state, the maximum corrosion depth of the 7A36 aluminum alloy with added Sc and an applied low-frequency electromagnetic field decreased from 113.6 μm to 49.1 μm.

## Figures and Tables

**Table 1:** Chemical composition of the studied alloys (wt.%) [23].

Alloy	Zn	Mg	Cu	Zr	Mn	Ti	Si	Fe	Sc	Al
A1	11.413	2.593	1.092	0.138	0.006	0.015	0.245	0.014	-	Bal
A2	11.620	2.501	1.070	0.129	0.005	0.010	0.247	0.013	0.012	Bal
A3	11.350	2.571	1.041	0.130	0.005	0.010	0.250	0.013	0.013	Bal

## Data Availability

The original contributions presented in this study are included in the article. Further inquiries can be directed to the corresponding authors.
